# Opioid utilization among pediatric patients treated for newly diagnosed acute myeloid leukemia

**DOI:** 10.1371/journal.pone.0192529

**Published:** 2018-02-08

**Authors:** Kelly D. Getz, Tamara P. Miller, Alix E. Seif, Yimei Li, Yuan-Shung V. Huang, Brian T. Fisher, Richard Aplenc

**Affiliations:** 1 Division of Oncology, The Children’s Hospital of Philadelphia, Philadelphia, Pennsylvania, United States of America; 2 Center for Pediatric Clinical Effectiveness, The Children’s Hospital of Philadelphia, Philadelphia, Pennsylvania, United States of America; 3 Department of Pediatrics, University of Pennsylvania School of Medicine, Philadelphia, Pennsylvania, United States of America; 4 Center for Clinical Epidemiology and Biostatistics, Unive of Pennsylvania School of Medicine, Philadelphia, Pennsylvania, United States of America; 5 Division of Infectious Diseases, The Children’s Hospital of Philadelphia, Philadelphia, Pennsylvania, United States of America; Queen's University Belfast, UNITED KINGDOM

## Abstract

**Purpose:**

A cohort of pediatric patients with AML treated at hospitals contributing to the Pediatric Health Information System was used to evaluate differences in opioid utilization by sex, age, race, and insurance.

**Methods:**

Billing data were used to compute the prevalence of opioid exposure and to quantify rates of utilization among those exposed to opioids as days of use per 1000 inpatient days. Multivariable regressions were used to compare opioid prevalence, and rates of utilization among those exposed.

**Results:**

On average across courses, 95.2% of patients were exposed to analgesics, 84.7% were exposed to non-opioid analgesics and 77.7% were exposed to opioids. The proportion of opioid-exposed patients increased with age, but did not differ by gender, race, or insurance status. Analyses limited to patients exposed to opioids revealed modest differences in days of opioid use among female patients (adjusted rate ratio (aRR) = 1.19, 95% CI: 1.11, 1.28), patients <1 year (aRR = 1.37, 95% CI: 1.21, 1.55) or ≥10 years of age (aRR = 1.63, 95% CI: 1.46, 1.82), whereas Asian patients received fewer days of opioids compared with white patients (aRR = 0.76, 95% CI: 0.61, 0.95). There was moderate hospital-level variability in both the prevalence of opioid utilization overall and preference for specific opioid medications. There was greater inconsistency in practice concerning choices for supplemental and alternative opioids than in first-line opioid utilization.

**Conclusion:**

Additional work is needed to discern whether observed differences in opioid utilization by age and race reflect a difference in treatment or a difference in the experience of pain. Future studies should also explore the factors which guide decisions on opioid selections in an attempt to explain the variability across institutions.

## Introduction

Pain is the most common symptom experienced by pediatric patients with cancer [1, 2]. Pain may be caused by malignancy, but is more commonly due to adverse effects of chemotherapy or invasive procedures [3]. Based on adult data, one-third of cancer patients present with pain at diagnosis and nearly 90% report pain at some time during therapy [4, 5]. Pain can negatively affect quality of life [6–9], result in patient and family distress [10, 11], and is associated with long-term morbidity [12]. Thus, effective pain management is a vital aspect of oncology patient care.

Opioids are recognized by WHO as essential for treatment of moderate-to-severe pain in children and are the mainstay of oncology pain management [13]. While 70–90% of cancer-related pain can be controlled by medication [14, 15], clinical and patient barriers may lead to suboptimal treatment. Poor pain assessment by provider, patient reluctance to report pain, patient and physician concerns about dependence, and inadequate knowledge on pain management are consistently reported in adult literature as the primary impediments to successful pain control [16]. Similar barriers to pain management have been identified for adolescents with cancer; however, unique barriers including fear about parental reactions have also been reported [17]. Because of the subjective nature of pain, successful management relies on effective communication between patients and providers. In pediatric populations, barriers may be further complicated due to an inability of patients to communicate their pain and because communications regarding illness, its treatment and subsequent side effects involve not only physician and patient, but also parents and caregivers. These barriers to effective pain assessment and communication may disproportionately affect certain subpopulations leading to differences in pain control.

Differences in opioid prescribing by patient race and socioeconomic status have been reported for a variety of diagnoses among adult and pediatric patients in emergency department and ambulatory care settings with a majority of studies finding non-white patients and uninsured patients less likely to receive opioids [18–25]. Studies of hospitalized pediatric patients have reported that opioid exposure prevalence was higher for females, older children, and white children [21, 22, 26, 27]. Others have also identified significant institutional variation in the prevalence and length of opioid use [27, 28]. However, the previous studies included a heterogeneous mix of malignant and nonmalignant diagnoses which likely have different levels of objectivity regarding the source of pain and variable profiles with respect to pain intensity and duration, and thus variability in the pharmaceutical approach to pain control.

Objectives of the current study were to describe overall trends in opioid utilization among a homogeneous population of pediatric patients undergoing standard chemotherapy treatment for newly diagnosed acute myeloid leukemia and to evaluate whether differences in opioid utilization exist with respect to sex, age, race, or insurance status. Hospital-level variation in opioid utilization was also assessed.

## Methods

### Data source

The Pediatric Health Information System (PHIS) is an administrative database that contains inpatient, emergency department, ambulatory surgery, and observation unit information from over 40 not-for-profit, tertiary care pediatric hospitals representing 17 large metropolitan areas in the United States [29]. Data include demographics, service dates, discharge disposition, and daily inpatient billing for medications, laboratory tests, imaging, clinical services, and supplies. Patients are assigned a unique identifier allowing records to be linked across admissions. Data are anonymized at submission and subject to reliability and validity checks before inclusion in the database. Quality is assured through a joint effort between the Children’s Hospital Association and participating hospitals.

### Study population

The current study population was derived from a cohort of children receiving chemotherapy for new onset AML assembled from PHIS data using a previously described and validated process [30]. Briefly, PHIS data from 2000–2014 were first screened for index admissions with a discharge diagnosis for any myeloid or unspecified leukemia (ICD-9-CM codes 205.xx–208.xx). Those with a diagnosis for an alternative malignancy or an indication of bone marrow transplantation within 60 days after the first diagnosis admission were excluded. Daily pharmacy data for each patient were then manually reviewed and chemotherapy administration patterns were matched to conventional pediatric AML treatment regimens. To further establish a uniform population of patients with new onset AML, the final cohort was restricted to patients who received standard chemotherapy defined as a match to the following course-specific regimens: ADE (cytarabine, daunorubicin, etoposide) at Induction I and Induction II, AE (cytarabine, etoposide) at Intensification I, MA (mitoxantrone, cytarabine) at Intensification II, and high-dose cytarabine with L-asparaginase (Capizzi schedule) at Intensification III. If a patient received chemotherapy that was inconsistent with the regimens defined above, that course and all subsequent courses were excluded from analyses.

### Outcome

Course-specific follow-up began on the first inpatient day of systemic chemotherapy and continued until the earliest of death, start of the next chemotherapy course, or 30 days (Inductions I and II), 35 days (Intensifications I and II) or 40 days (Intensification III) after commencement of that chemotherapy course. Course-specific follow-up periods were based on experience regarding expected time to absolute neutrophil count recovery.

The primary outcome of interest was opioid exposure. Opioids included morphine, fentanyl, oxycodone, hydromorphone, meperidine, methadone, codeine, alfentanil, remifentanil, nalbuphine, butorphanol, sufentanil, levorphanol, oxymorphone, and pentazocine. We first evaluated the occurrence of opioid exposure (yes, no), overall (exposure to any one of the specific opioids itemized above, regardless of route of administration) and for specific agents, during the course-specific follow-up periods. Then, we assessed the rates of utilization among the subset of patients receiving opioids. To compute the rates of utilization among those exposed, binary indicator variables were created for each agent to designate exposure on each inpatient day which were summed to obtain the total number of days exposed. Opioid utilization rates were reported as the number of days of use per 1000 inpatient days. Information on the utilization of non-opioid analgesics (i.e., acetaminophen, ibuprofen, naproxen, celecoxib) was also similarly summarized. Given that the chemotherapy regimens utilized in frontline treatment for AML in pediatric patients in the United States are not associated with neuropathy, adjuvant pain medications are generally not utilized and are therefore not included in this analysis. Of note, PHIS data do not include information on medication dosage or frequency and does not include non-pharmacological treatments for pain.

### Covariates

Patient characteristics including gender, race as recorded by the treating institution (categorized as white, black, Asian, and other), age (categorized as <1, 1 to <5, 5 to <10, 10 to <15, and ≥15 years), insurance status at the start of each course as recorded by the treating institution (categorized as private, public, and other), and diagnosis year were ascertained from PHIS.

ICU-level care was captured at each course as a marker for patients with a more severe clinical status and potentially higher pain intensity. ICU-level care was defined by the occurrence of specific ICD-9-CM procedure codes or clinical resources considered a priori as a marker of ICU care, rather than by physical location [31]. The predominant cause of treatment-related pain in this patient population is mucositis [2] which could present with varying degrees of severity and not warrant ICU-level care, but affect oral intake requiring initiation of parenteral nutritional support. Thus, we also captured total parenteral nutrition requirements (TPN) at each course. ICU-level care and TPN were included in analyses to control for confounding by severity/indication.

### Statistical analyses

Distributions of patient demographic and clinical characteristics were tabulated by course and compared across courses using chi-square tests. Opioid exposure prevalence, overall and for specific agents, was computed for each course. Trends in opioid exposure prevalence over time were evaluated using chi-square tests. Daily prevalence estimates for specific opioids were plotted to illustrate trends in utilization over each chemotherapy course. Multivariable log-binomial regression models were used to estimate adjusted prevalence ratios (aPR) and corresponding 95% confidence intervals (CI) comparing the prevalence of analgesic use overall, and separately for opioid use and non-opioid use, by covariates defined above. Among those exposed to the class, multivariable Poisson regression models with inpatient days as offset were used to estimate adjusted rate ratios (aRR) and 95% CI comparing opioid utilization rates. Robust variance estimates were obtained using generalized estimating equation methods with an exchangeable correlation matrix to account for intra-individual correlation between course-specific observations. Fully adjusted models included all demographic and clinical factors examined. Patients with missing covariate information were excluded from analyses.

Plots of hospital-specific opioid prevalence and rates of use among those exposed as well as a plot of the proportion of hospitals reporting each specific opioid were generated to assess variability in practice. Pearson correlations (*R*) were used to identify associations between the two hospital-level opioid utilization variables and their association with the total number of AML cases contributed per year.

All statistical analyses were performed using SAS (version 9.2, SAS Institute, Inc., Cary, NC).

### Human subject protections

This study was deemed by the Institutional Review Board of the Children’s Hospital of Philadelphia to not constitute human subjects research.

## Results

### Study population

The initial PHIS cohort included 1,695 patients with newly diagnosed AML treated at 42 institutions across the United States. Of these, approximately 94% (n = 1,600) met inclusion criteria for at least one course and in total contributed 4,902 chemotherapy courses (S1 Fig).

Distributions of patient characteristics by chemotherapy course are presented in Table 1. Despite attrition at each successive course, there were no differences in course-specific distributions of gender, age, race or insurance status. However, there was significant variability in TPN and ICU-level care requirements between specific chemotherapy courses (p<0.0001). TPN requirements were similar during Induction I and Intensification II (31% versus 27%) and significantly higher compared with requirements during other courses (17–22%). The largest difference in ICU-level care was observed between Induction I and Induction II (16% vs. 4%; p<0.0001). Across intensification courses, there was less variability in ICU-level care requirements (7–11%; p = 0.122).

**Table 1:** Patient characteristics by chemotherapy course.

		Induction I (N = 1600)	Induction II (n = 1249)	Intensification I (n = 990)	Intensification II (n = 671)	Intensification III (n = 392)	
		%	%	%	%)	%)	p-value
Gender							0.9119
	Male	52.3	52.2	50.8	50.8	52.6	
	Female	47.7	47.8	49.2	49.2	47.4	
Age, years							0.5271
	<1	11.7	12.2	11.8	11.6	12.5	
	1 to < 5	24.7	25.9	26.3	28.8	31.9	
	5 to <10	17.0	16.3	16.6	16.2	14.8	
	10 to <15	26.0	26.4	26.3	25.8	25.5	
	15 to <20	20.6	19.3	19.1	17.6	15.3	
Race							0.9788
	White	70.6	71.4	71.5	73.0	73.2	
	Black	13.5	12.8	12.7	11.5	13.3	
	Asian	3.9	3.7	3.3	3.6	2.8	
	Other	11.9	12.1	12.4	11.9	10.7	
Insurance							0.9901
	Private	41.9	42.0	42.0	43.2	41.1	
	Public	42.7	41.5	41.8	41.7	42.3	
	Other	15.4	16.6	16.2	15.1	16.6	
Diagnosis Year							<0.0001
	2000–2001	0.3	0.3	0.0	0.0	0.0	
	2002–2003	1.8	2.3	0.9	0.4	0.0	
	2004–2005	16.1	17.5	15.9	15.2	22.2	
	2006–2007	18.7	20.0	20.5	22.1	28.8	
	2008–2009	20.8	22.3	23.6	21.8	28.3	
	2010–2011	21.6	21.1	21.6	21.6	19.4	
	2012–2014	22.9	19.0	18.3	18.9	1.3	
Parenteral Nutrition		31.0	16.8	17.2	27.6	22.7	<0.0001
ICU level care		15.9	4.4	7.2	11.2	10.5	<0.0001

### Trends in opioid utilization

**Fig 1:**
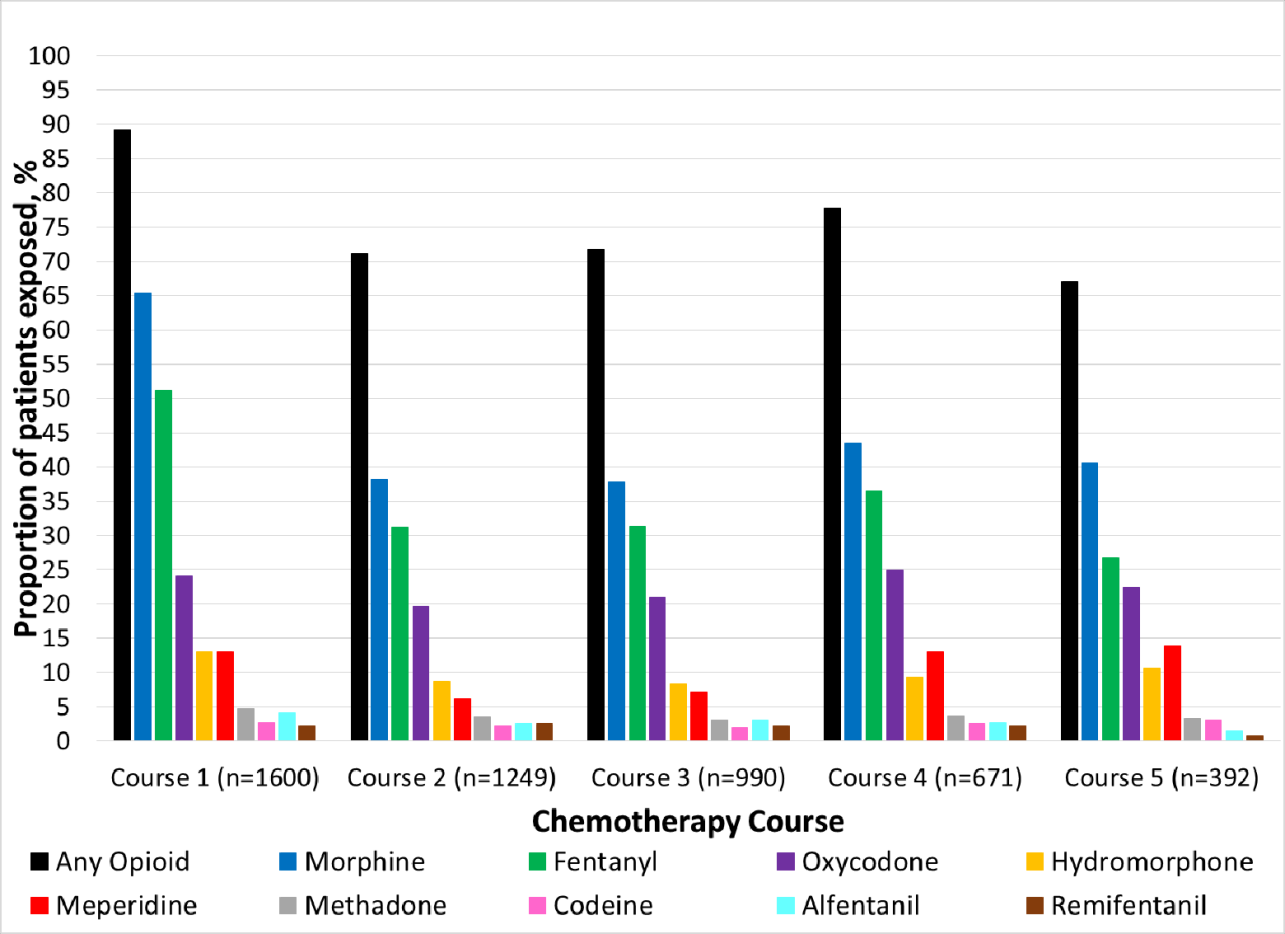
Prevalence of opioid utilization among pediatric AML patients by chemotherapy course.

Overall, 95.2% of courses were exposed to analgesics, 84.7% were exposed to non-opioid analgesics (mainly acetaminophen) and 77.7% were exposed to opioids. Intravenous (68.8%) opioid administration was more common than oral administration (44.5%) (S1 Table). The pattern of course-specific opioid utilization was similar to that observed for ICU-level care with Induction I having the highest prevalence followed by Intensification II. While the overall prevalence of opioid use was higher for Induction I than other chemotherapy courses (89.2% versus 71.9%, aPR = 1.23, 95% CI: 1.19, 1.26), the relative frequencies of utilization for specific opioid medications were generally similar across courses (Fig 1 and S1 Table). The most prevalent opioids were morphine, fentanyl and oxycodone with 47.9%, 38.2% and 22.6% of patients being exposed on average across courses, respectively; the remaining opioids were utilized by 10% or fewer patients across courses.

Overall there was a temporal trend toward reduced opioid utilization with 88.9% of courses exposed in 2000–2001 and 73.9% of courses exposed in 2012–2014 (p = 0.041) and corresponding increase in non-opioid utilization (70.0% versus 88.2%, p<0.001). The decline in opioids was primarily driven by significant declines in codeine (5.7% of courses in 2002–2003 to 0.2% of courses in 2012–2014, p<0.001) and meperidine utilization (21.4% of courses in 2002–2003 to 5.2% of courses in 2012–2014, p<0.001).

**Fig 2:**
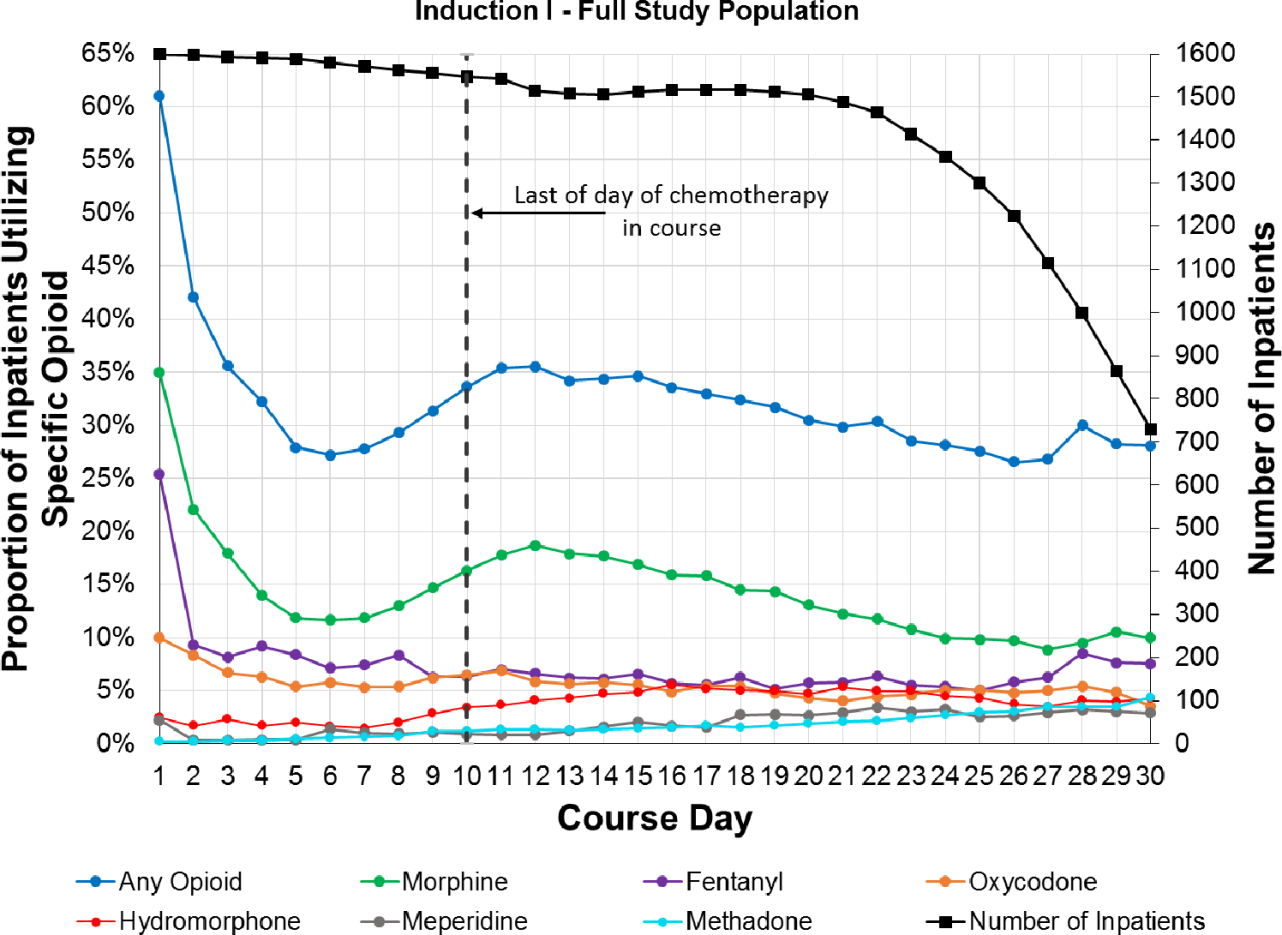
Patterns of daily inpatient utilization of specific opioids over Induction I.

Fig 2 presents the patterns of daily inpatient utilization of opioids, overall and for specific agents, during Induction I. There were two distinct peaks in overall prevalence of opioid utilization. The first occurred at the start of the course and the second after completion of course-specific chemotherapy. This overall trend was driven by trends in the most commonly utilized opioid, morphine. The prevalence of fentanyl use was highest at course start and declined to a consistent prevalence rate by the completion of course-specific chemotherapy. The prevalence of oxycodone utilization was generally consistent over the duration of the course. Exposure to the infrequently utilized opioids such as hydromorphone, meperidine and methadone was more common after chemotherapy completion. Patterns of opioid utilization observed for subsequent courses were generally similar to Induction I, but with lower overall exposure prevalence (S2 Fig, Panels a-d).

### Comparisons of utilization by patient characteristics

Table 2 and S2 Table present adjusted comparisons of the prevalence of opioid and non-opioid exposure overall and for the most commonly used specific opioid medications, respectively. Opioid exposure overall was more common among patients older than 10 years; this trend was observed for the specific opioids morphine and oxycodone, but not for fentanyl. ICU level care (aPR = 1.10, 95% CI: 1.07, 1.14) and the requirement for TPN (aPR = 1.16, 95% CI: 1.13, 1.20) were each associated with a higher prevalence of opioid use overall. Increases were reflective primarily of increases in morphine use among patients requiring TPN (aPR = 1.43, 95% CI: 1.33, 1.55) and fentanyl use among those requiring ICU-level care (aPR = 1.61, 95% CI: 1.39, 1.87). There were no significant differences in opioid exposure prevalence by gender, race, or insurance. Similar trends were observed for the prevalence of non-opioid utilization.

**Table 2:** Multivariable adjusted comparisons of opioid exposure prevalence.

		Opioid Prevalence, %	Opioid Prevalence Ratio (95% confidence interval)	Non-opioid Prevalence, %	Non-opioid Prevalence Ratio (95% confidence interval)
Gender					
	Female	81.3	0.99 (0.96, 1.01)	84.8	1.01 (0.99, 1.03)
	Male	82.2	Reference	84.7	Reference
Age, years					
	<1	78.2	1.06 (0.99, 1.14)	82.7	1.02 (0.96, 1.08)
	1 to < 5	73.8	Reference	79.0	Reference
	5 to <10	78.1	1.06 (0.99, 1.12)	86.5	1.07 (1.02, 1.13)*
	10 to <15	87.2	1.18 (1.11, 1.26)*	87.7	1.11 (1.05, 1.18)*
	15 to <20	92.9	1.26 (1.18, 1.34)*	88.4	1.12 (1.04, 1.20)*
Race					
	White	79.8	Reference	85.3	Reference
	Black	81.9	1.03 (0.98, 1.08)	80.7	1.00 (0.96, 1.04)
	Asian	82.6	1.03 (0.93, 1.16)	93.2	1.06 (1.00, 1.11)
	Other	82.6	1.03 (0.99, 1.08)	83.5	1.02 (0.94, 1.11)
Insurance					
	Private	80.9	Reference	84.6	Reference
	Public	82.2	1.01 (0.98, 1.05)	82.8	1.00 (0.96, 1.03)
	Other	82.1	1.01 (0.96, 1.07)	90.1	1.04 (0.98, 1.12)
Total Parenteral Nutrition					
	Yes	88.1	1.16 (1.13, 1.20)*	88.4	1.06 (1.01, 1.11)*
	No	75.8	Reference	83.6	Reference
ICU level care					
	Yes	85.9	1.10 (1.07, 1.14)*	92.1	1.04 (1.00, 1.09)
	No	77.8	Reference	83.9	Reference

*p-value <0.05

All models adjusted for presented covariates, chemotherapy course and diagnosis year.

Comparisons of the rates of opioid use among those exposed (Table 3) revealed more days of use among female patients (aRR = 1.19, 95% CI: 1.11, 1.28). Stratification revealed that the association with gender differed by age. While there was an increase in the rate of opiate use among exposed females over 10 years of age (aRR = 1.29, 95% CI: 1.18, 1.42), there was no such increase among females aged 10 years or younger (aRR = 1.01, 95% CI: 0.90, 1.14). Exposed patients <1 year of age (aRR = 1.37, 95% CI: 1.21, 1.55) and ≥10 years of age (aRR = 1.63, 95% CI: 1.46, 1.82) also experienced more days of opioid use compared to those 1 to <10 years of age, whereas Asian patients received fewer days of opioids compared with white patients (aRR = 0.76, 95% CI: 0.61, 0.95). Trends in the rates of use for the commonly used specific opioids were generally similar to those observed for opioids overall (S3 Table).

**Table 3:** Multivariable adjusted comparisons of the rates of opioid and non-opioid utilization among exposed patients.

		Opioid Rate^a^	Opioid Rate Ratio (95% CI)	Non-opioid Rate^a^	Non-opioid Rate Ratio (95% CI)
Gender					
	Female	327.1	1.19 (1.11, 1.28)*	340.9	0.99 (0.93, 1.04)
	Male	274.5	Reference	345.3	Reference
Age, years					
	<1	301.8	1.37 (1.21, 1.55)*	275.0	0.93 (0.85, 1.02)
	1 to < 5	209.3	Reference	295.1	Reference
	5 to <10	234.0	1.04 (0.91, 1.17)	365.6	1.24 (1.12, 1.37)
	10 to <15	319.8	1.42 (1.26, 1.59)*	390.5	1.32 (1.20, 1.46)
	15 to <20	445.4	1.84 (1.66, 2.03)*	410.2	1.39 (1.24, 1.56)
Race					
	White	333.4	Reference	347.5	Reference
	Black	312.1	0.94 (0.85, 1.03)	327.8	0.94 (0.87, 1.02)
	Asian	255.4	0.76 (0.61, 0.95)*	345.4	0.99 (0.90, 1.10)
	Other	303.4	0.91 (0.82, 1.01)	352.1	1.01 (0.96, 1.07)
Insurance					
	Private	292.4	Reference	328.2	Reference
	Public	294.2	1.01 (0.93, 1.09)	330.0	1.00 (0.94, 1.08)
	Other	312.8	1.07 (0.98, 1.16)	372.8	1.11 (0.98, 1.24)
Total Parenteral Nutrition					
	Yes	358.2	1.43 (1.30, 1.57)*	394.5	1.32 (1.22, 1.43)
	No	250.7	Reference	298.4	Reference
ICU level care					
	Yes	367.8	1.51 (1.39, 1.64)*	389.8	1.29 (1.23, 1.36)
	No	244.1	Reference	302.0	Reference

^a^Rate reported as number of days of use per 1000 inpatient days

*p-value <0.05

All models adjusted for presented covariates, chemotherapy course and diagnosis year.

Rates of non-opioid utilization among those exposed increased with age (p<0.001). Otherwise, trend in the rates of utilization paralleled those observed for the overall prevalence of non-opioid use.

### Hospital-level variability

**Fig 3:**
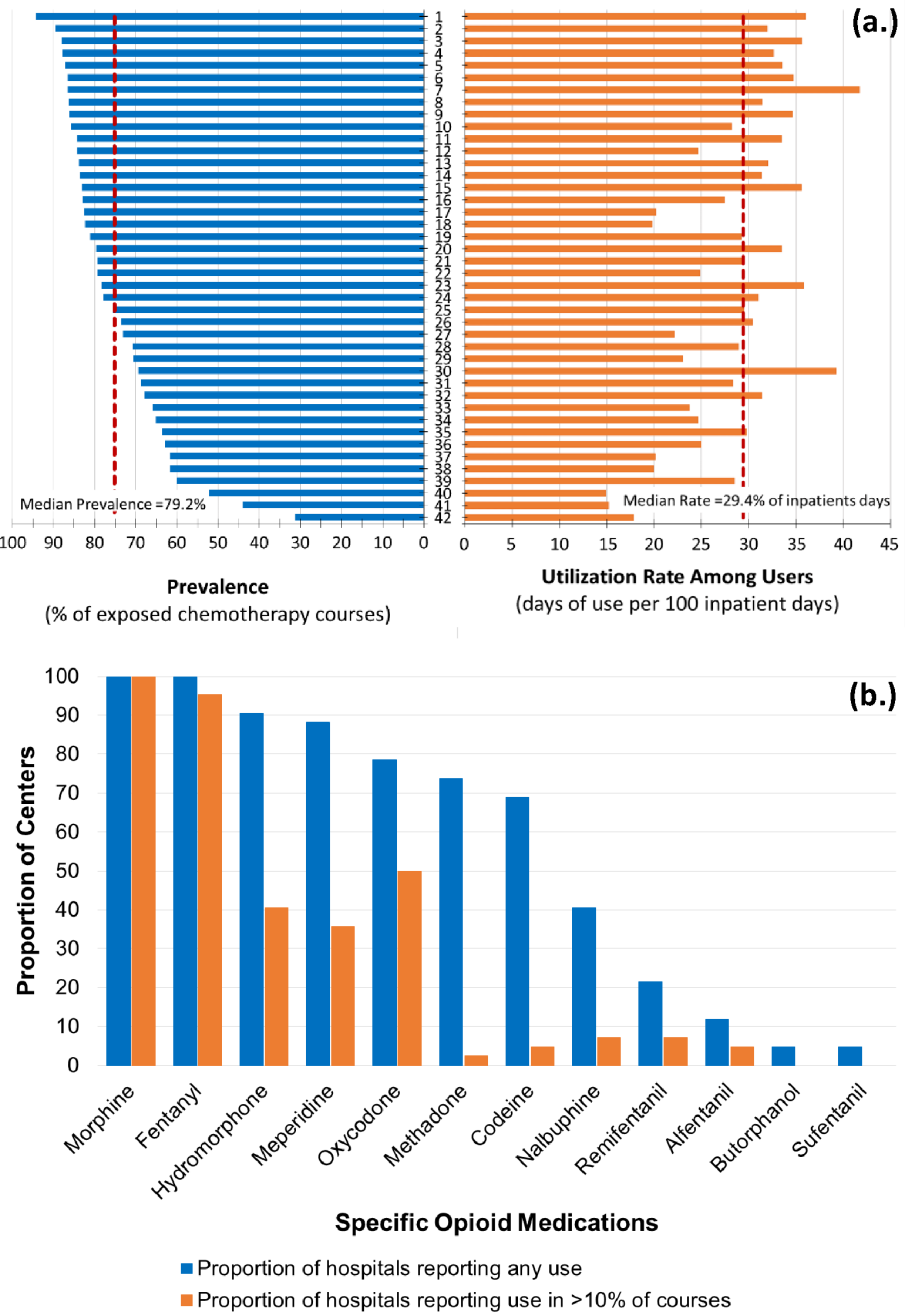
**Center-level variation in (a) the prevalence and rate of overall opioid utilization and (b) the use of specific opioid medications** In (a), hospitals are numbered in order of decreasing prevalence of opioid use.

Across hospitals, there was moderate variability in the proportion of courses exposed to opioids (IQR: 67.7–84.3%, p<0.001) as well as the average rate of utilization among opioid-exposed patients (IQR: 246–334 days of use per 1000 inpatient days; p<0.001) (Fig 3, Panel a). Higher hospital-level opioid exposure prevalence correlated with higher average number of inpatient days of use (*R* = 0.661, p<0.001). Additionally, higher total number of AML diagnoses per year was moderately correlated with both higher hospital-level opioid prevalence (*R* = 0.369, p = 0.016) and higher average rate of utilization among those exposed (*R* = 0.352, p = 0.022). There was no significant correlation between hospital-level opioid and non-opioid utilization with respect to prevalence (p = 0.174) or averages number of inpatient days of use (0.077).

There was also variability across hospitals in the use of specific opioids (Fig 3, Panel b). Only morphine and fentanyl were consistently utilized by all hospitals in the study. The remaining opioid medications were utilized by 5–90% of institutions, and if used, they were reported in <10% of courses at the majority of institutions.

## Discussion

Our study found that the likelihood of opioid exposure among pediatric patients with newly diagnosed AML varied by treatment course and consistently differed by age. Among those exposed to opioids, there were only modest differences in the total number of days of use by gender and race. In addition, we observed moderate hospital-level variability in both opioid utilization overall and preference for specific opioid medications. There was greater inconsistency in practice concerning choices for supplemental and alternative opioids than in first-line opioid utilization. Together these findings provide some reassurance that large disparities in pain control in pediatric AML patients are unlikely.

Opioid utilization was highest in Induction I and Intensification II which likely reflects higher patient acuity at diagnosis, additional invasive diagnostic evaluations, and greater treatment-related complications in those courses, an explanation supported by the parallel increase in ICU-level care and TPN utilization. Patterns of daily inpatient utilization of specific opioids observed in the current study are consistent with expectations based on guidelines for use in practice, thus highlighting the overall validity of the data resource. In particular, the bimodal distribution of morphine and the single peak of fentanyl at the start of a chemotherapy course reflect their predominant uses as first-line pain management and procedural pain control, respectively. Patterns of use observed for other specific opioids accurately correspond to their use as supplemental pain management or as alternative treatment in patients who experience morphine-related adverse effects.

Observed differences in opioid utilization by age and Asian race are comparable to prior reports. One previous study using a general population of pediatric inpatients reported that opioid exposure was lowest among infants and increased with age but once exposed infants experience a longer duration of use [28]. Others have also identified a higher prevalence of undertreated pain among Asian cancer patients [18]. Differences in clinical evaluation and patient reporting of pain may explain some of the observed differences in opioid use by age and Asian race [16–18, 32, 33]. Alternatively, it possible that the observed differences in utilizaton may also reflect differences in the occurrence or intensity of pain.

Our finding that opioid-exposed female patients experienced more days of opioids compared to males, though small, is also consistent with others who reported higher sensitivity to both clinically and experimentally induced pain, higher pain scores, and greater temporal summation of pain in females [26, 33–36]. The specific underlying mechanisms contributing to this difference are not clear. However, given that the gender differences in opioid use were restricted to patients older than 10 years provides some support for the theory that the differences may be influenced by hormonal factors which may vary between prepubertal and adolescent patients, with testosterone being more protective than estradiol [34, 36–38]. While observed differences in opioid utilization between male and female patients may reflect differences in biologic thresholds for the experience of pain, they may equally support the role of gender schema theory translating to differences in the threshold for reporting pain, pain coping styles, and parental behaviour in response to pain [37].

In contrast to other studies on opioid prescribing for a variety of diagnoses in emergency department and ambulatory care settings [18–25]particularly for with subjective sources of pain (e.g. back pain) than objective sources (e.g., long bone fracture) [21], we did not observe a difference in inpatient opioid utilization between white and black patients. In the case of cancer pain management, it may be that there is less partiality around the occurrence of pain and the requirement for pain control and thus less opportunity for differential prescribing.

We found that higher hospital volume of AML diagnoses correlated with both higher hospital-level opioid prevalence and longer average duration of use among exposed patients. A higher volume of AML diagnoses may be a proxy for greater experience in its treatment, thus differences clinical experience may be a contributing factor to the observed institutional variation in opioid utilization.

Our analyses were performed using a large nationally representative cohort of pediatric AML patients, which enhances the generalizability of our results. Also, unlike most previous evaluations of opioid use in hospitalized pediatric populations which include a combination of diagnoses, our study population was restricted to a homogeneous cohort of newly diagnosed AML patients receiving the same standard chemotherapy treatment. In the absence of this restriction, any heterogeneity in the associations between patient factors and opioid use across diagnoses would be obscured with results reflecting the associations for the most common diagnoses.

Our results should be considered in light of potential limitations. First, PHIS does not include medication dosage or frequency data thus precluding the evaluation of these factors. As such, it is possible that the observed center-level variability may reflect differences in institutional prescribing trends. For example, an institution that administers opioids daily could be administering less total opioid than a center which uses continuous dosing but on fewer days. PHIS does not capture results of pain assessments thus we have no information on the levels of either pain or pain relief. We also lack data on the exact indication prompting utilization of pharmacologic agents and non-pharmacologic strategies employed to relieve pain. Therefore, we cannot differentiate whether observed differences in opioid utilization between patients reflect differences in pain assessment, pain management, or the severity of pain experienced. Likewise, the lack of an observable difference in opioid use between some compared groups may not reflect equivalent adequacy of pain management particularly if there are differences in acuity which we did not have the ability to directly measure. We attempted to adjust for differences in severity of pain using ICU-level care and TPN requirements as a crude proxy to identify patients with more severe pain. However, observed differences in opioid use were not explained by differences in TPN or ICU utilization. Lastly, these results only reflect trends in inpatient opioid utilization and may not extend to trends in outpatient analgesia.

In summary, these data suggest that patient-level and hospital-level variability exists in the utilization of opioids during the treatment of pediatric AML. The limitations of our data resource prevent definitive conclusions regarding the reason for such differences, but suggest areas for further research. Namely, additional work is needed to identify the specific mechanisms leading to these differences and to address ways to overcome treatment barriers and mitigate disparities in pain management, if such inequities exist. Future studies should focus on identifying the factors which guide decisions on opioid selections in an attempt to explain the wide variability across institutions. Research efforts aimed at refining pain prevention and developing best practices and clinical guidelines for optimal pain assessments and treatment are necessary to maximize standardization in pain management in pediatric AML.

## Supporting information

S1 Table Frequency (n, %) of non-opioid and opioid use among pediatric patients treated for newly diagnosed acute myeloid leukemia, overall and by chemotherapy course. (DOCX)

S2 Table Multivariable adjusted comparisons of the prevalence of exposure to common specific opioid medications among AML patients by gender, age, race, insurance, parental nutrition requirements, and ICU level care requirements. (DOCX)

S3 Table Multivariable adjusted comparisons of the rate of utilization (days of use per 100 inpatient days) of common specific opioid medications by gender, age, race, insurance, parental nutrition requirements, and ICU level care requirements among AML patients exposed to opioid medications. (DOCX)

S1 Fig Assembly of study population from established PHIS cohort of pediatric patients with acute myeloid leukemia. Abbreviations: PHIS = Pediatric Health Information System database; AML = acute myeloid leukemia, pt = patients.
(DOCX)

S2 Fig Patterns of daily inpatient utilization of specific opioids over (a) Induction II, (b) Intensification I, (c) Intensification II, (d) Intensification III.
(DOCX)
