# Supplementary material for: Opioid utilization among pediatric patients treated for newly diagnosed acute myeloid leukemia
Source: PLoS One. 2018 Feb 8;13(2):e0192529. doi: 10.1371/journal.pone.0192529 (PMC5805309; doi:10.1371/journal.pone.0192529)
Supplement: S1 Table — (DOCX) [file pone.0192529.s001.docx]

**S1 Table. Frequency (n, %) of non-opioid and opioid use among pediatric patients treated for newly diagnosed acute myeloid leukemia, overall and by chemotherapy course**

|  |  |  | **Across Courses (n=4902)** | **Induction I (n=1600)** | **Induction II (n=1249)** | **Intensification I (n=990)** | **Intensification II (n=671)** | **Intensification III (n=392)** |
| --- | --- | --- | --- | --- | --- | --- | --- | --- |
|  |  |  | n (%) | n (%) | n (%) | n (%) | n (%) | n (%) |
| **Any Analgesia^a^** | | | 4665 (95.2) | 1575 (98.4) | 1148 (91.9) | 923 (93.2) | 651 (97.0) | 368 (93.9) |
| **Any non-opioid^b^** | | | 4154 (84.7) | 1414 (88.4) | 992 (79.4) | 813 (82.1) | 594 (88.5) | 341 (87.0) |
| Acetaminpphen | | | 4123 (84.1) | 1409 (88.1) | 985 (78.9) | 806 (81.4) | 584 (87.0) | 339 (86.5) |
| Ibuprofen | | | 275 (5.6) | 77 (4.8) | 58 (4.6) | 69 (7.0) | 58 (8.6) | 13 (3.3) |
| Celecoxib | | | 116 (2.4) | 29 (1.8) | 23 (1.8) | 27 (2.7) | 23 (3.4) | 14 (3.6) |
| Naproxen | | | 28 (0.57) | 5 (0.3) | 5 (0.4) | 7 (0.7) | 6 (0.9) | 5 (1.3) |
| **Any opioid^c^** | | | 3811 (77.7) | 1427 (89.2) | 889 (71.2) | 710 (71.7) | 522 (77.8) | 263 (67.1) |
|  | Morphine | | 2349 (47.9) | 1046 (65.4) | 477 (38.2) | 375 (37.9) | 292 (43.5) | 159 (40.6) |
|  | Fentanyl | | 1871 (38.2) | 819 (51.2) | 391 (31.3) | 311 (31.4) | 245 (36.5) | 105 (26.8) |
|  | Oxycodone | | 1109 (22.6) | 401 (25.1) | 245 (19.6) | 208 (21.0) | 167 (24.9) | 88 (22.4) |
|  | Hydromorphone | | 505 (10.3) | 209 (13.1) | 109 (8.7) | 82 (8.3) | 63 (9.4) | 42 (10.7) |
|  | Meperidine | | 498 (10.2) | 210 (13.1) | 77 (6.2) | 70 (7.1) | 87 (13.0) | 54 (13.8) |
|  | Methadone | | 188 (3.8) | 76 (4.8) | 45 (3.6) | 29 (3) | 25 (3.7) | 13 (3.3) |
|  | Codeine | | 167 (3.4) | 82 (5.1) | 31 (2.5) | 30 (3) | 18 (2.7) | 6 (1.5) |
|  | Alfentanil | | 117 (2.4) | 41 (2.6) | 27 (2.2) | 20 (2) | 17 (2.5) | 12 (3.1) |
|  | Remifentanil | | 107 (2.2) | 35 (2.2) | 31 (2.5) | 23 (2.3) | 15 (2.2) | 3 (0.8) |
|  | Nalbuphine | | 91 (1.9) | 46 (2.9) | 18 (1.4) | 10 (1.0) | 12 (1.8) | 5 (1.3) |
|  | Butorphanol | | 5 (0.1) | 3 (0.2) | 0 (0) | 0 (0) | 0 (0) | 2 (0.5) |
|  | Sufentanil | | 3 (0.1) | 1 (0.1) | 1 (0.1) | 0 (0) | 1 (0.1) | 0 (0) |
|  | **Any parenteral opioid** | | 3374 (68.8) | 1342 (83.9) | 748 (59.9) | 600 (60.6) | 456 (68.0) | 228 (58.2) |
|  |  | Morphine | 2305 (47.0) | 1040 (65.0) | 459 (36.7) | 367 (37.1) | 283 (42.2) | 156 (39.8) |
|  |  | Fentanyl | 1856 (37.9) | 816 (51.0) | 385 (30.8) | 307 (31.0) | 244 (36.4) | 104 (26.5) |
|  |  | Meperidine | 495 (10.1) | 209 (13.1) | 76 (6.1) | 69 (7.0) | 87 (13.0) | 54 (13.8) |
|  |  | Hydromorphone | 452 (9.2) | 198 (12.4) | 91 (7.3) | 66 (6.7) | 56 (8.3) | 41 (10.5) |
|  |  | Alfentanil | 117 (2.4) | 41 (2.6) | 27 (2.2) | 20 (2) | 17 (2.5) | 12 (3.1) |
|  |  | Remifentanil | 107 (2.2) | 35 (2.2) | 31 (2.5) | 23 (2.3) | 15 (2.2) | 3 (0.8) |
|  |  | Nalbuphine | 91 (1.9) | 46 (2.9) | 18 (1.4) | 10 (1.0) | 12 (1.8) | 5 (1.3) |
|  |  | Methadone | 78 (1.6) | 41 (2.6) | 14 (1.1) | 8 (0.8) | 8 (1.2) | 7 (1.8) |
|  |  | Butorphanol | 5 (0.1) | 3 (0.2) | 0 (0) | 0 (0) | 0 (0) | 2 (0.5) |
|  |  | Sufentanil | 3 (0.1) | 1 (0.1) | 1 (0.1) | 0 (0) | 1 (0.1) | 0 (0) |
|  |  | Codeine | 1 (0) | 1 (0.1) | 0 (0) | 0 (0) | 0 (0) | 0 (0) |
|  | **Any oral opioid** | | 2179 (44.5) | 821 (51.3) | 492 (39.4) | 386 (39.0) | 310 (46.2) | 170 (43.4) |
|  |  | Oxycodone | 1109 (22.6) | 401 (25.1) | 245 (19.6) | 208 (21.0) | 167 (24.9) | 88 (22.4) |
|  |  | Morphine | 213 (4.3) | 73 (4.6) | 46 (3.7) | 39 (3.9) | 35 (5.2) | 20 (5.1) |
|  |  | Methadone | 160 (3.3) | 59 (3.7) | 41 (3.3) | 26 (2.6) | 22 (3.3) | 12 (3.1) |
|  |  | Hydromorphone | 141 (2.9) | 42 (2.6) | 35 (2.8) | 33 (3.3) | 23 (3.4) | 8 (2.0) |
|  |  | Codeine | 167 (3.4) | 81 (5.1) | 31 (2.5) | 30 (3) | 18 (2.7) | 6 (1.5) |
|  |  | Fentanyl | 5 (0.1) | 2 (0.1) | 2 (0.2) | 1 (0.1) | 0 (0) | 0 (0) |
|  |  | Meperidine | 6 (0.1) | 2 (0.1) | 1 (0.1) | 1 (0.1) | 1 (0.1) | 1 (0.3) |

*exposure to any one of the opioids evaluated in our analyses, regardless of route of administration
