# Supplementary material for: Opioid utilization among pediatric patients treated for newly diagnosed acute myeloid leukemia
Source: PLoS One. 2018 Feb 8;13(2):e0192529. doi: 10.1371/journal.pone.0192529 (PMC5805309; doi:10.1371/journal.pone.0192529)
Supplement: S2 Table — (DOCX) [file pone.0192529.s002.docx]

**S2 Table. Multivariable adjusted comparisons of the prevalence of exposure to common specific opioid medications among AML patients by gender, age, race, insurance, parental nutrition requirements, and ICU level care requirements**

|  |  | **Morphine** | | **Fentanyl** | | **Oxycodone** | |
| --- | --- | --- | --- | --- | --- | --- | --- |
|  | | Prevalence, % | PR (95% CI) | Prevalence, % | PR (95% CI) | Prevalence, % | PR (95% CI) |
| Gender | |  |  |  |  |  |  |
|  | Female | 49.4 | 0.99 (0.93, 1.05) | 40.5 | 0.98 (0.91, 1.05) | 20.1 | 1.10 (0.98, 1.22) |
|  | Male | 49.8 | 1 (reference) | 41.6 | 1 (reference) | 18.2 | 1 (reference) |
| Age at diagnosis | |  |  |  |  |  |  |
|  | <1 year | 47.7 | 1.11 (0.99, 1.26) | 41.7 | 1.08 (0.95, 1.22) | 17.0 | 1.33 (1.05, 1.69)* |
|  | 1 to < 5 years | 42.6 | 1 (reference) | 38.7 | 1 (reference) | 12.8 | 1 (reference) |
|  | 5 to <10 years | 47.4 | 1.11 (0.98, 1.22) | 39.1 | 1.01 (0.91, 1.12) | 17.3 | 1.36 (0.95, 1.94) |
|  | 10 to <15 years | 52.7 | 1.24 (1.13, 1.35)* | 42.4 | 1.09 (0.98, 1.22) | 22.1 | 1.73 (1.31, 2.29)* |
|  | 15 to <20 years | 59.1 | 1.39 (1.26, 1.53)* | 43.6 | 1.12 (0.99, 1.26) | 30.9 | 2.42 (1.75, 3.39)* |
| Race | |  |  |  |  |  |  |
|  | White | 49.5 | 1 (reference) | 41.4 | 1 (reference) | 18.3 | 1 (reference) |
|  | Black | 47.5 | 0.96 (0.87, 1.05) | 45.2 | 1.09 (0.99, 1.20) | 18.6 | 1.02 (0.91, 1.13) |
|  | Asian | 47.1 | 0.95 (0.79, 1.14) | 39.7 | 0.96 (0.74, 1.24) | 19.4 | 1.06 (0.83, 1.35) |
|  | Other | 54.6 | 1.10 (0.99, 1.21) | 38.3 | 0.92 (0.83, 1.03) | 20.3 | 1.11 (0.99, 1.24) |
| Insurance | |  |  |  |  |  |  |
|  | Private | 50.2 | 1 (reference) | 39.7 | 1 (reference) | 18.1 | 1 (reference) |
|  | Public | 49.7 | 0.99 (0.92, 1.06) | 41.8 | 1.05 (0.97, 1.14) | 19.9 | 1.10 (0.96, 1.26) |
|  | Other | 48.8 | 0.97 (0.85, 1.11) | 41.7 | 1.05 (0.89, 1.23) | 19.4 | 1.07 (0.84, 1.36) |
| Parenteral Nutrition | |  |  |  |  |  |  |
|  | Yes | 59.4 | 1.43 (1.33, 1.55)* | 46.2 | 1.26 (1.16, 1.38)* | 19.2 | 1.00 (0.89, 1.13) |
|  | No | 41.4 | 1 (reference) | 36.5 | 1 (reference) | 19.1 | 1 (reference) |
| ICU level care | |  |  |  |  |  |  |
|  | Yes | 54.4 | 1.20 (1.10, 1.32)* | 52.1 | 1.61 (1.39, 1.87)* | 18.1 | 0.90 (0.77, 1.05) |
|  | No | 45.2 | 1 (reference) | 32.3 | 1 (reference) | 20.2 | 1 (reference) |
| *p-value <0.05  All models adjusted for each of the other presented covariates, chemotherapy course and diagnosis year. | | | | | | | |
