# Supplementary material for: Opioid utilization among pediatric patients treated for newly diagnosed acute myeloid leukemia
Source: PLoS One. 2018 Feb 8;13(2):e0192529. doi: 10.1371/journal.pone.0192529 (PMC5805309; doi:10.1371/journal.pone.0192529)
Supplement: S3 Table — (DOCX) [file pone.0192529.s003.docx]

**S3 Table. Multivariable adjusted comparisons of the rate of utilization (days of use per 100 inpatient days) of common specific opioid medications by gender, age, race, insurance, parental nutrition requirements, and ICU level care requirements among AML patients exposed to opioid medications**

|  |  | **Morphine** | | **Fentanyl** | | **Oxycodone** | |
| --- | --- | --- | --- | --- | --- | --- | --- |
|  | | Rate^a^ | PR (95% CI) | Rate^a^ | PR (95% CI) | Rate^a^ | PR (95% CI) |
| Gender | |  |  |  |  |  |  |
|  | Female | 204.3 | 1.17 (1.07, 1.28)* | 94.7 | 1.15 (0.97, 1.36) | 170.8 | 1.15 (1.02, 1.29)* |
|  | Male | 174.4 | 1 (reference) | 82.5 | 1 (reference) | 148.7 | 1 (reference) |
| Age at diagnosis | |  |  |  |  |  |  |
|  | <1 year | 205.6 | 1.43 (1.25, 1.62)* | 91.6 | 1.19 (0.99, 1.50) | 234.3 | 1.83 (1.54, 2.18)* |
|  | 1 to < 5 years | 144.2 | 1 (reference) | 76.4 | 1 (reference) | 127.9 | 1 (reference) |
|  | 5 to <10 years | 162.2 | 1.12 (0.99, 1.27) | 76.6 | 1.00 (0.70, 1.43) | 116.1 | 0.91 (0.78, 1.06) |
|  | 10 to <15 years | 203.0 | 1.41 (1.23, 1.61)* | 102.7 | 1.35 (1.00, 1.83)* | 148.7 | 1.16 (0.98, 1.37) |
|  | 15 to <20 years | 245.4 | 1.70 (1.51, 1.91)* | 97.9 | 1.28 (1.02, 1.61)* | 198.8 | 1.55 (1.29, 1.87)* |
| Race | |  |  |  |  |  |  |
|  | White | 208.7 | 1 (reference) | 101.1 | 1 (reference) | 164.7 | 1 (reference) |
|  | Black | 188.7 | 0.91 (0.83, 1.01) | 123.7 | 1.22 (1.00, 1.47) | 172.4 | 1.05 (0.85, 1.29) |
|  | Asian | 158.2 | 0.76 (0.56, 1.03) | 57.9 | 0.57 (0.30, 1.11) | 138.3 | 0.83 (0.59, 1.19) |
|  | Other | 184.1 | 0.88 (0.76, 1.03) | 84.3 | 0.83 (0.53, 1.30) | 164.5 | 1.00 (0.81, 1.23) |
| Insurance | |  |  |  |  |  |  |
|  | Private | 190.1 | 1 (reference) | 88.2 | 1 (reference) | 163.8 | 1 (reference) |
|  | Public | 178.9 | 0.94 (0.84, 1.06) | 82.9 | 0.94 (0.84, 1.06) | 179.1 | 1.09 (0.95, 1.26) |
|  | Other | 197.8 | 1.04 (0.92, 1.17) | 94.5 | 1.07 (0.87, 1.32) | 138.0 | 0.85 (0.74, 1.00) |
| Parenteral Nutrition | |  |  |  |  |  |  |
|  | Yes | 220.8 | 1.37 (1.23, 1.53)* | 98.9 | 1.25 (1.04, 1.51)* | 161.4 | 1.02 (0.93, 1.13) |
|  | No | 161.3 | 1 (reference) | 79.0 | 1 (reference) | 157.4 | 1 (reference) |
| ICU level care | |  |  |  |  |  |  |
|  | Yes | 215.3 | 1.30 (1.15, 1.47)* | 142.9 | 2.73 (1.94, 3.52)* | 149.6 | 0.88 (0.72, 1.08) |
|  | No | 165.4 | 1 (reference) | 54.7 | 1 (reference) | 169.9 | 1 (reference) |
| ^a^expressed as days of use per 1000 hospital days; *p-value <0.05 | | | | | | | |
| All models adjusted for each of the other presented covariates, chemotherapy course and diagnosis year. | | | | | | | |
