# Supplementary material for: Opioid utilization among pediatric patients treated for newly diagnosed acute myeloid leukemia
Source: PLoS One. 2018 Feb 8;13(2):e0192529. doi: 10.1371/journal.pone.0192529 (PMC5805309; doi:10.1371/journal.pone.0192529)
Supplement: S1 Fig — Abbreviations: PHIS = Pediatric Health Information System database; AML = acute myeloid leukemia, pt = patients. (DOCX) [file pone.0192529.s004.docx]

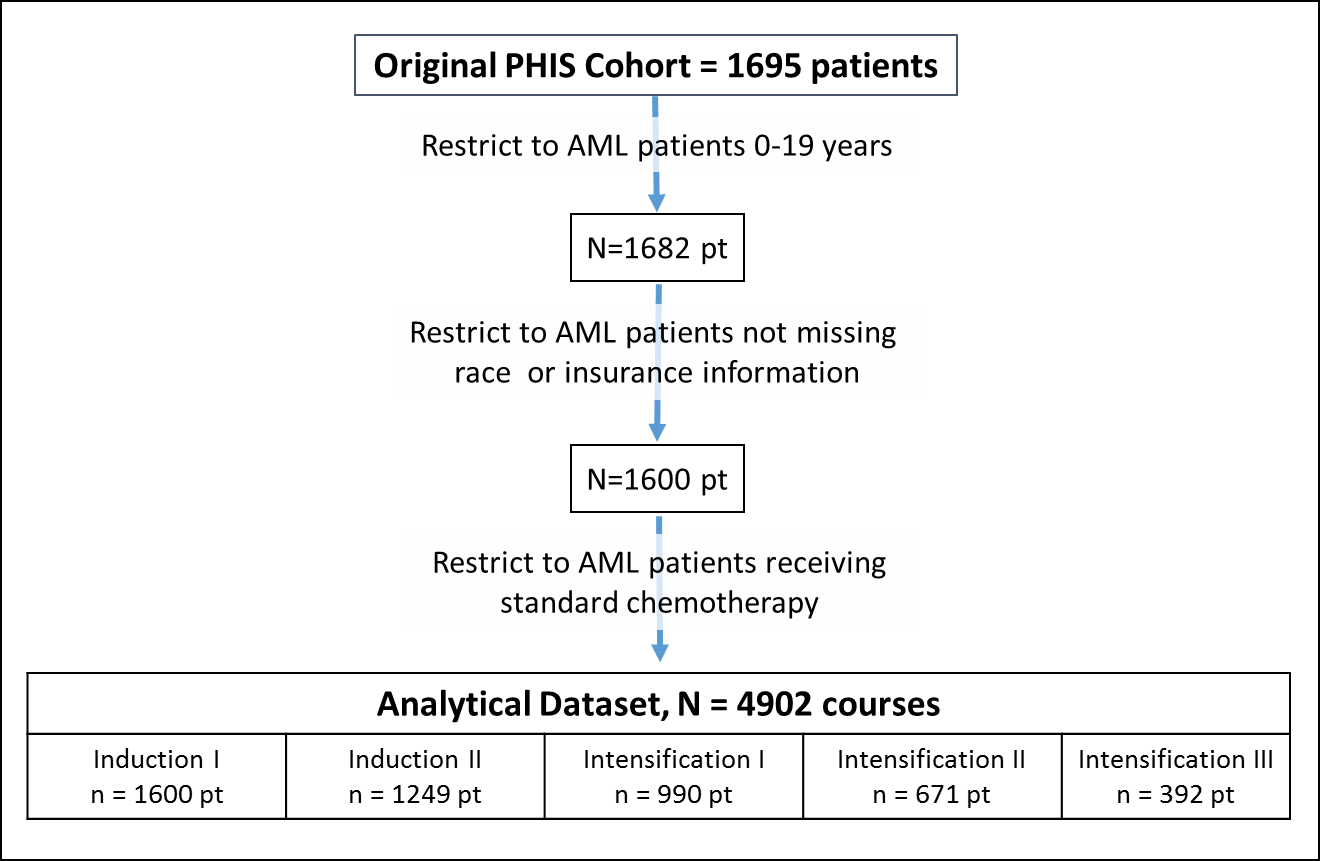


**S1 Fig. Assembly of study population from established PHIS cohort of pediatric patients with acute myeloid leukemia** Abbreviations: PHIS=Pediatric Health Information System database; AML=acute myeloid leukemia, pt=patients
