# Supplementary material for: Opioid utilization among pediatric patients treated for newly diagnosed acute myeloid leukemia
Source: PLoS One. 2018 Feb 8;13(2):e0192529. doi: 10.1371/journal.pone.0192529 (PMC5805309; doi:10.1371/journal.pone.0192529)
Supplement: S2 Fig — Patterns of daily inpatient utilization of specific opioids over (a) Induction II, (b) Intensification I, (c) Intensification II, (d) Intensification III. (DOCX) [file pone.0192529.s005.docx]

**a.)**

**b.)**


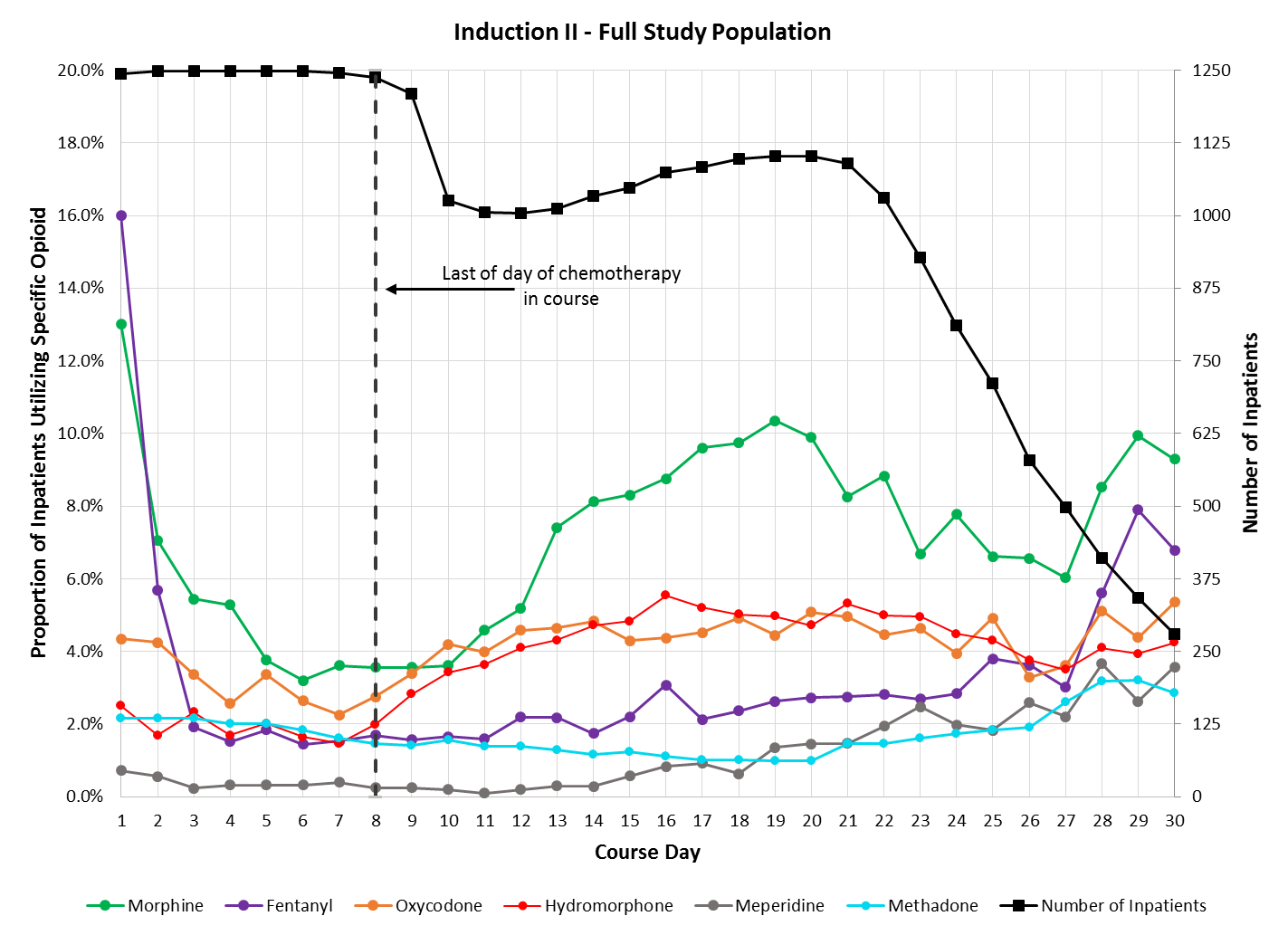

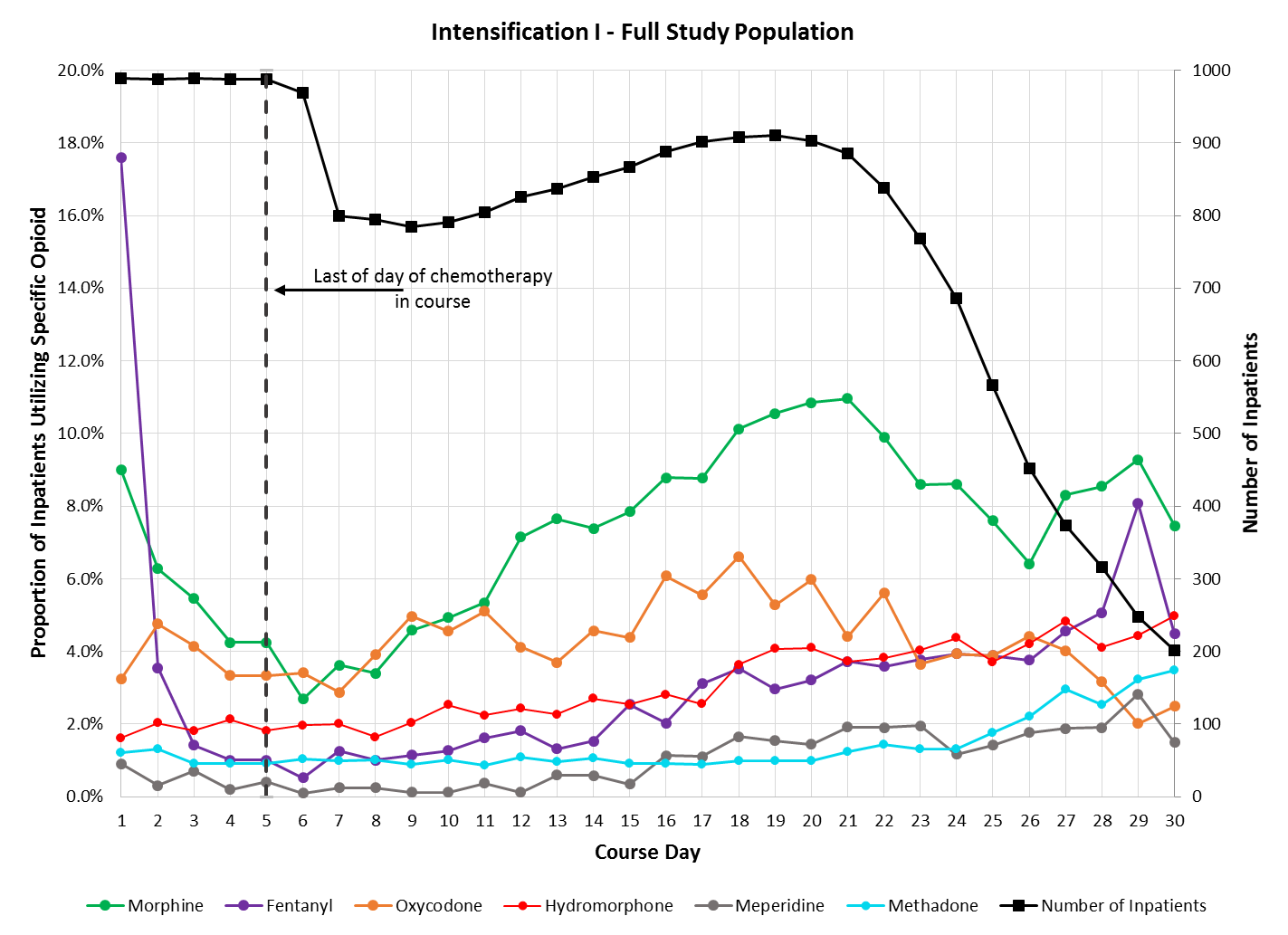


**d.)**

**c.)**


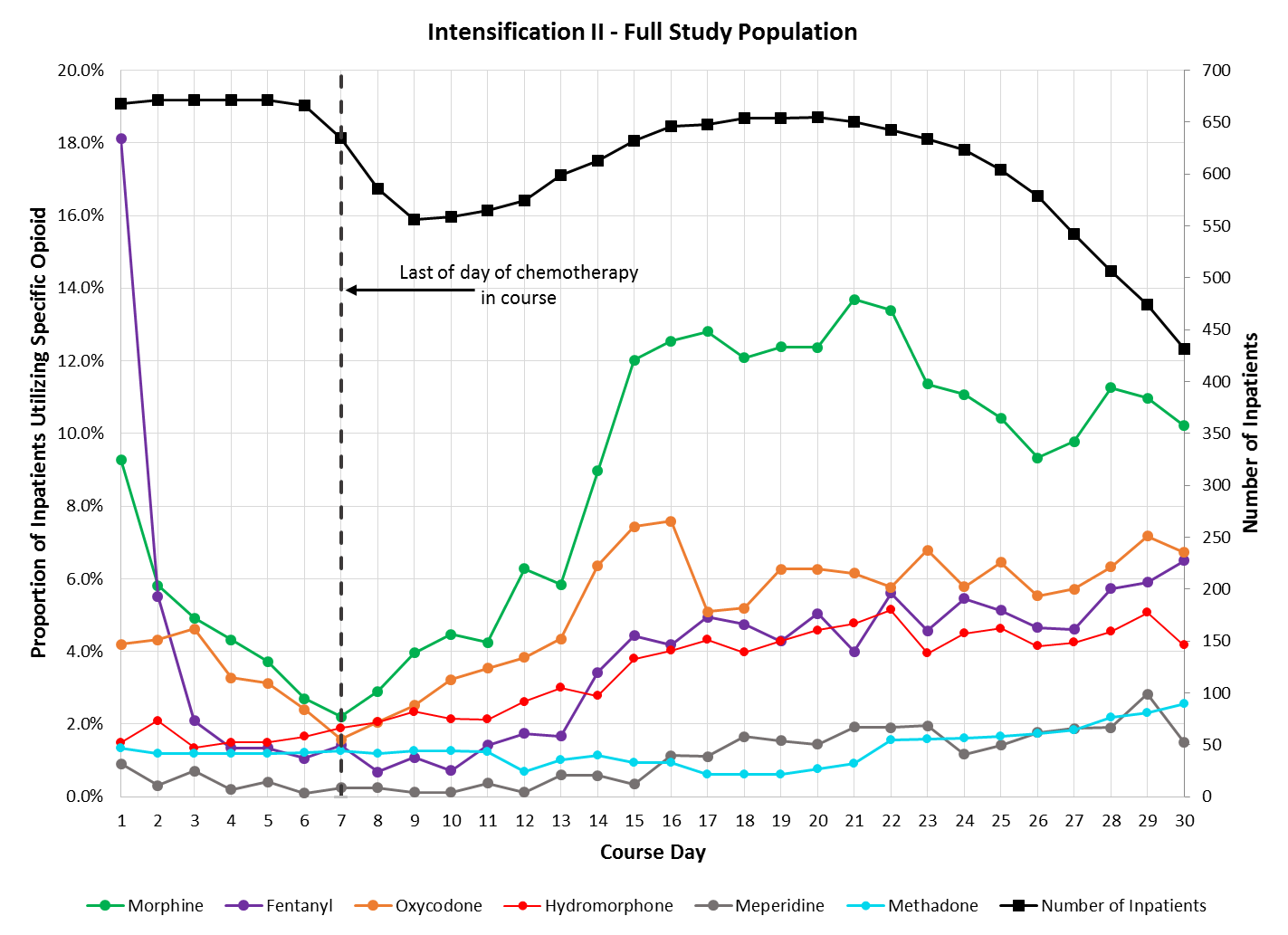

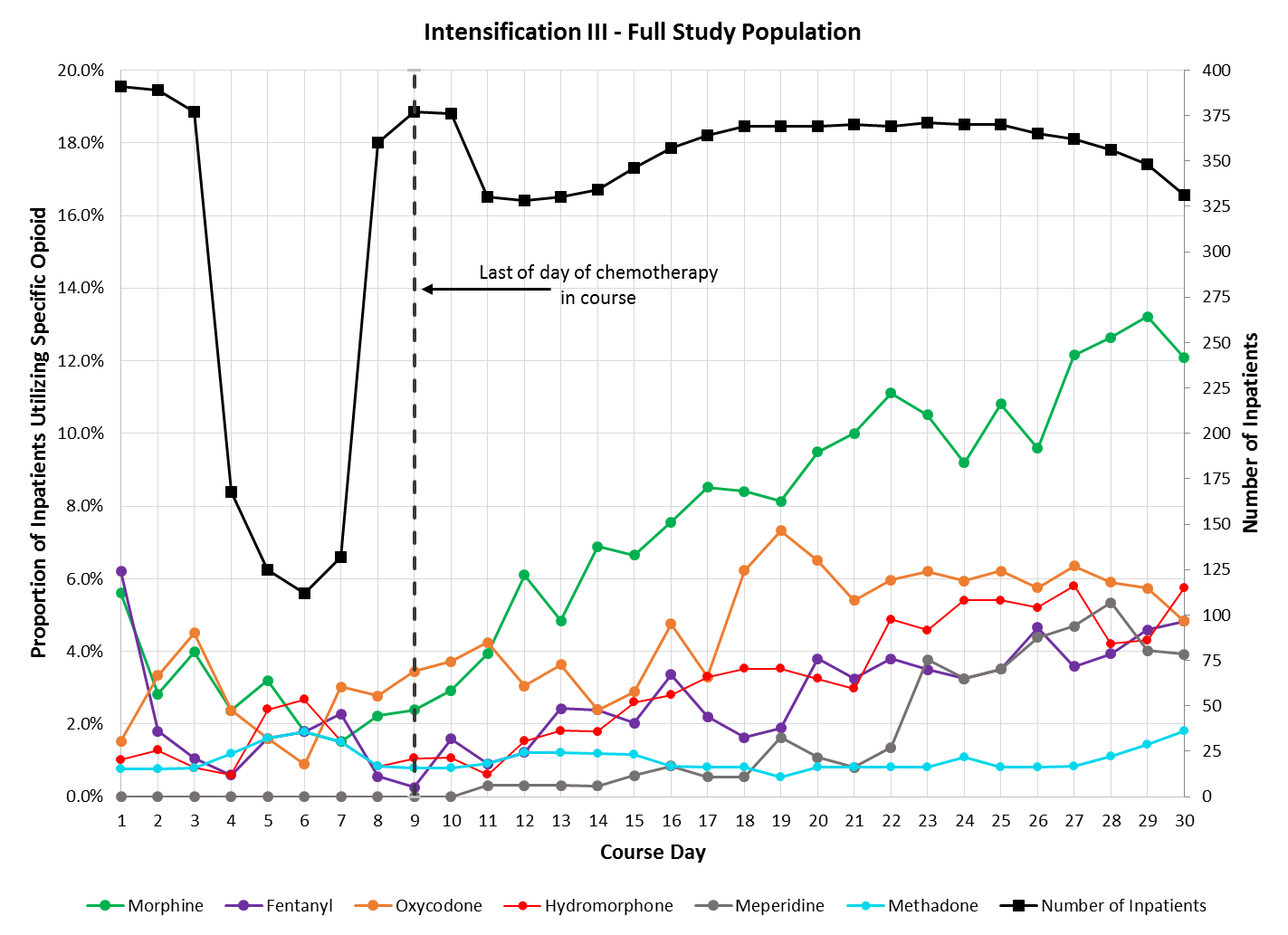


**S2 Fig. Patterns of daily inpatient utilization of specific opioids over (a) Induction II, (b) Intensification I, (c) Intensification II, (d) Intensification III**
